# Design of Composite N-Doped Carbon Nanofiber/TiO_2_/Diatomite Separator for Lithium–Sulfur Batteries

**DOI:** 10.3390/ma17225615

**Published:** 2024-11-17

**Authors:** Wenjie Xiao, Xiaoyu Wu, Yang Shu, Yitao Zha, Sainan Liu

**Affiliations:** 1 School of Minerals Processing and Bioengineering, Central South University, Changsha 410083, China; xiaowenjie9574@163.com (W.X.);; 2 School of Materials Science and Engineering, Central South University, Changsha 410083, China

**Keywords:** lithium–sulfur batteries, electrospinning and carbonization, carbon nanofiber, titanium dioxide, diatomite

## Abstract

Lithium–sulfur batteries (LSBs) exhibit high theoretical specific capacities, abundant resource reserves, and low costs, making them promising candidates for next-generation lithium-ion batteries (LIBs). However, significant challenges, such as the shuttle effect and volume expansion, hinder their practical applications. To address these issues, this study introduces a unique intermediate layer comprising N-doped carbon nanofiber/TiO_2_/diatomite (NCNF/TiO_2_/DE) from the perspective of membrane modification. The intermediate layer comprises nitrogen-doped titanium dioxide/carbon nanofiber (NCNF/TiO_2_) materials, with diatomite filling the fiber gaps. This forms a three-dimensional (3D) conductive network that provides ample space for sulfur volume expansion and numerous adsorption active sites, thereby accelerating electrolyte penetration and lithium-ion diffusion. These features collectively contribute to the outstanding electrochemical performance of the battery. At 0.1 C, the NCNF/TiO_2_/DE-800-coated separator battery achieved a first-cycle discharge specific capacity of 1311.1 mAh g^−1^, significantly higher than the uncoated lithium–sulfur battery (919.6 mAh g^−1^). Under varying current densities, the NCNF/TiO_2_/DE-800 material demonstrates good electrochemical reversibility and exhibits high lithium-ion diffusion rates and low charge-transfer resistance. Therefore, this study provides an advanced intermediate layer material that enhances the electrochemical performance of lithium–sulfur batteries.

## 1. Introduction

Since the turn of the century, the rapid development of the new energy industry has spurred extensive research into high-performance energy storage batteries [1,2,3]. Lithium–sulfur (Li–S) secondary batteries, constructed with lithium metal as the negative electrode, and sulfur as the positive electrode, possess a high theoretical specific capacity (1675 mAh g^−1^) and specific energy (2600 Wh kg^−1^). Sulfur, as an environmentally benign element, is abundant and cost-effective, making Li–S batteries one of the ideal choices for the next generation of electrochemical energy storage systems [4,5]. However, lithium–sulfur batteries still face several challenges, primarily including: (1) the poor conductivity of sulfur and its discharge products, which reduce battery transmission efficiency; (2) the multi-phase, multi-step conversion of sulfur within the battery, characterized by slow kinetics, leads to the “shuttle” of polysulfides between the positive and negative electrodes, continuously depleting active materials and degrading electrochemical performance; (3) significant volume expansion during charge and discharge, which severely impacts the practical application of lithium–sulfur batteries [6,7,8].

Researchers have proposed various strategies to address these issues, such as cathode design [9,10,11], electrolyte modification [12,13], and traditional separator modification [14,15,16]. Separators serve as channels for lithium-ion transport and effectively prevent short circuits [17]. However, in most cases, polysulfides pass through traditional separators and react directly with the lithium anode, leading to electrode passivation, capacity loss, an increase in impedance, and the degradation of cycling performance [18]. Modifying traditional separators with conductive functional materials, such as intermediate layers, is an effective strategy to improve battery performance by inhibiting the “shuttle effect” and reducing corrosion of the lithium anode [19]. Recently, intermediate layer materials with polysulfide-capturing capabilities have garnered significant interest from researchers. For example, carbon-based materials, such as carbon nanofibers and graphene, are extensively studied due to their high conductivity and physical adsorption of polysulfides [20,21]. However, the interaction between polar polysulfides and non-polar carbon-based materials primarily occurs via van der Waals forces, which significantly limits their effectiveness in improving cycling performance. Researchers have found that doping carbon-based materials with heteroatoms, such as oxygen, sulfur, and nitrogen, can alter the polarity of the materials to some extent [22,23]. Polar oxides (such as Al_2_O_3_ [24], SiO_2_ [25], and CeO_2_ [26]) strongly interact with polysulfides, effectively accelerating their conversion and enhancing the performance of lithium–sulfur batteries [27,28]. For example, researchers have prepared modified porous carbon materials using gelatin as the base material and nickel nitrate as the template, through processes such as dehydration foaming, KOH, and HCl etching, which exhibit good electrochemical performance in lithium–sulfur batteries [29]. Despite these advancements, designing the microstructure of intermediate layers to achieve effective polysulfide conversion remains a challenge. Of the polar oxides, TiO_2_ is considered a promising material due to its excellent chemical stability and catalytic activity toward polysulfides [30]. Diatomite, with its rich porous structure and numerous Si-OH groups [31], can serve as an excellent filler for carbon-based materials, enhancing their physical adsorption capability and accelerating polysulfide conversion. However, the introduction of diatomite into intermediate layers has been rarely reported.

This study employed electrospinning, peroxidation, and carbonization processes to fabricate nitrogen-doped carbon nanofiber/titanium dioxide-filled diatomite (NCNF/TiO_2_/DE) as an intermediate layer for lithium–sulfur batteries. The intermediate layer consists of nitrogen-doped carbon fiber/titanium dioxide (NCNF/TiO_2_) materials, providing effective pathways for Li^+^ diffusion and improving the conductivity of the layer while mitigating sulfur volume expansion. Diatomite fills gaps in the 3D network, and its abundant porous structure and Si-OH groups provide numerous active sites for capturing polysulfides. The results indicate that under 0.1 C conditions, the lithium–sulfur battery with an NCNF/TiO_2_/DE-800-modified separator exhibits superior electrochemical performance, achieving a first-cycle discharge specific capacity of 1311.1 mAh g^−1^, in comparison to the unmodified separator lithium–sulfur battery, which has a first-cycle specific capacity of 919.6 mAh g^−1^. Additionally, the modified material demonstrated improved electrochemical performance metrics, including cycling performance, rate capability, impedance, and lithium-ion diffusion rates.

## 2. Experimental

### 2.1. Materials

The Celgard 2500 was supplied by Shanghai Saibo Chemical Co., Ltd. (Shanghai, China) Sublimed sulfur (AR, 99.5%), urea (AR, 99%), polyvinylpyrrolidone (PVP, AR, 99%), and ethanol (AR, water ≤ 0.3%) were obtained from Shanghai Aladdin Bio-Chem Technology Co., Ltd. (Shanghai, China) *N*-methylpyrrolidone (NMP, 99.9%), tetrabutyl titanate (AR, 99%), glacial acetic acid (AR, 99.5%), polyvinylidene fluoride (PVDF), and *N*,*N*-dimethylformamide (DMF, AR, 99.5%) were supplied by Shanghai Macklin Biochemical Co., Ltd. (Shanghai, China) Acetylene black (Acet), 1.0 mol LiTFSI in DOL:DME = 1:1 vol% with 1.0 wt% LiNO_3_ were provided by Dongguan Kede Technology Co., Ltd. (Dongguan, China) The diatomite was sourced from Changbai Mountain and treated with 2 M hydrochloric acid for 3 h, then rinsed to neutrality, dried, and stored for use.

### 2.2. Preparation of Separator Composite Materials

First, a precursor solution was prepared as follows: in a 10 mL reagent bottle, 6 mL of DMF, 1 mL of ethanol, and 2 mL of glacial acetic acid were added. While stirring, 2 mL of tetrabutyl titanate was slowly dripped in. After mixing uniformly, 0.1 g of diatomite, 0.4 g of urea, and 1.125 g of PVP (AR, 99%) were sequentially added. The 10 mL reagent bottle was sealed and stirred at room temperature for 24 h.

For electrospinning, a metal syringe needle with an inner diameter of 0.6 mm was used. A 10 mL syringe containing the prepared precursor solution was fixed to an electrospinning device. The injection speed was set to 0.2 mm min^−1^, and the needle diameter was 1.6 cm. The distance between the spinneret and the collector was set to 15 cm, and the equipment applied a voltage of 25 kV. Electrospun fibers were collected on silicone oil paper, ensuring that no droplets formed on the receiving plate to avoid affecting fiber morphology.

After electrospinning, the electrospun membrane was peeled off the silicone oil paper and dried at 60 °C for 2 h. The membrane was then preoxidized at 260 °C in an air atmosphere for 2 h and, subsequently, transferred to a tube furnace. The materials were heated at a rate of 5 °C min^−1^ to 600 °C, 700 °C, and 800 °C under an argon atmosphere and maintained at these temperatures for 2 h each. After cooling, the final products were named NCNF/TiO_2_/DE-600, NCNF/TiO_2_/DE-700, and NCNF/TiO_2_/DE-800, respectively.

### 2.3. Preparation of Cathode Materials

Sublimed sulfur (S) and acetylene black (Acet) were mixed in a mass ratio of 3:1, ground uniformly, and heat-treated at 155 °C for 24 h to obtain Acet@S. For cathode preparation, Acet@S was mixed with PVDF and acetylene black in a mass ratio of 8:1:1, and an appropriate amount of N-methyl-2-pyrrolidone (NMP) was added. The mixture was stirred magnetically for 24 h to form a homogeneous slurry. The slurry was then evenly coated onto aluminum foil, dried at 60 °C for 2 h, and cut into circular cathodes with a diameter of 13 mm. The sulfur loading on the electrodes was approximately 1.3 mg cm^−2^.

### 2.4. Li_2_S_6_ Adsorption Capacity Test

In an argon glovebox, sublimed sulfur and Li_2_S were weighed in a molar ratio of 5:1 and dissolved in the electrolyte (DOL/DME = 1:1 vol%) for 24 h to prepare a 5 mM Li_2_S_6_ solution. Each sample (NCNF/TiO_2_/DE-600, NCNF/TiO_2_/DE-700, NCNF/TiO_2_/DE-800) was diluted to 5 mM, and 3 mL aliquots were added to four separate vials. Then, 1 mg of each sample was added to the respective Li_2_S_6_ solutions, allowed to stand for 12 h, and the supernatant was analyzed using UV-Vis absorption spectroscopy.

### 2.5. Electrochemical Testing

Cathode disks (12 mm diameter) were assembled with lithium metal (anode), Celgard 2500 (separator), and the fabricated intermediate layer in an argon-filled glovebox to form CR2032 coin cells. The electrolyte was 1.0 mol LiTFSI in DOL:DME = 1:1 vol% with 1.0 wt% LiNO_3_, with approximately 20–25 μL per cell. Before electrochemical testing, the coin cells were rested overnight.

Charge–discharge performance and rate performance tests were conducted using a multi-channel tester (LANHE CT2001A, Wuhan Landian Electronics Co., Ltd., Wuhan, China). Cyclic voltammetry (CV) and electrochemical impedance spectroscopy (EIS) were performed using a CHI660E electrochemical workstation (Shanghai Chenhua Instrument Co., Ltd., Shanghai, China). The CV scans were conducted in the voltage range of 1.7 to 2.8 V at scan rates of 0.1 to 0.5 mV s^−1^. EIS measurements were carried out over a frequency range of 10 mHz to 100 kHz.

### 2.6. Material Characterization

The morphological and elemental composition of the samples were characterized using scanning electron microscopy (SEM, TESCAN MIRA3 LMH, TESCAN Group a.s, Brno, Czech Republic) and energy-dispersive X-ray spectroscopy (EDS, Ultim Max 80, Oxford Instrument Technology (Shanghai) Co., Ltd, Shanghai China). Transmission electron microscopy (TEM, JEM-F200, JEOL Japan Electronics Co., Ltd, Tokyo, Japan) and EDS were used to observe the microstructure, lattice spacing, and elemental distribution of the fiber materials at 200 kV. Raman spectra were obtained using a Thermo Fisher DXR3 spectrometer with a 514 nm laser. X-ray diffraction (XRD, Rigaku DX-2500, Rigaku, Tokyo, Japan) was used to analyze the crystal structure of the materials over a 2*θ* range of 5° to 80°. X-ray photoelectron spectroscopy (XPS, Thermo Fisher K-ALPHA, Thermo Fisher Scientific, Waltham, MA, USA) was used to analyze the chemical states of the elements, with the carbon peak (C 1s, 284.8 eV) used as a reference. Nitrogen adsorption/desorption isotherms were measured using a Micromeritics ASAP 2020 instrument (Micromeritics Instrument Corp, Atlanta, GA, USA). Before measurement at −196 °C (liquid nitrogen), the samples were degassed under vacuum at 160 °C for 8 h.

## 3. Results and Discussion

**Figure 1:**
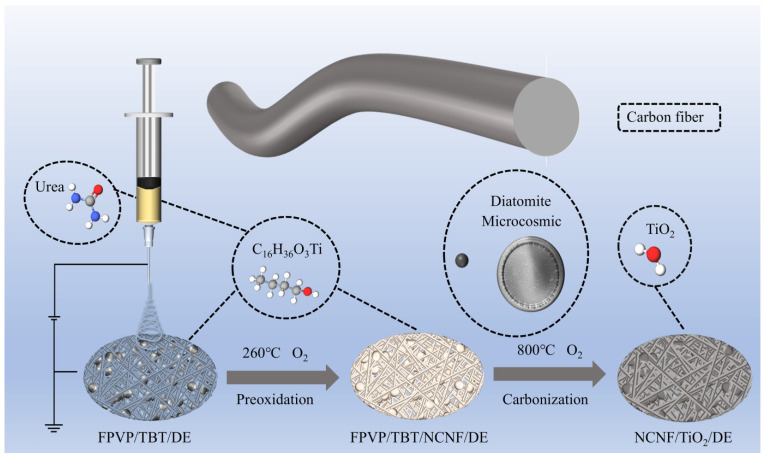
Schematic illustration of the synthesis process of NCNF/TiO_2_/DE.

As shown in Figure 1, a composite N-doped carbon nanofiber/TiO_2_/diatomite (NCNF/TiO_2_/DE) separator was fabricated using a combination of electrospinning, preoxidation, and carbonization. Initially, FPVP/C_10_H_14_O_5_Ti/DE (FPVP/TBT/DE) nanofiber membranes were obtained through electrospinning, followed by peroxidation, and carbonization at 800 °C to successfully transform them into the composite NCNF/TiO_2_/DE-800 separator.

**Figure 2:**
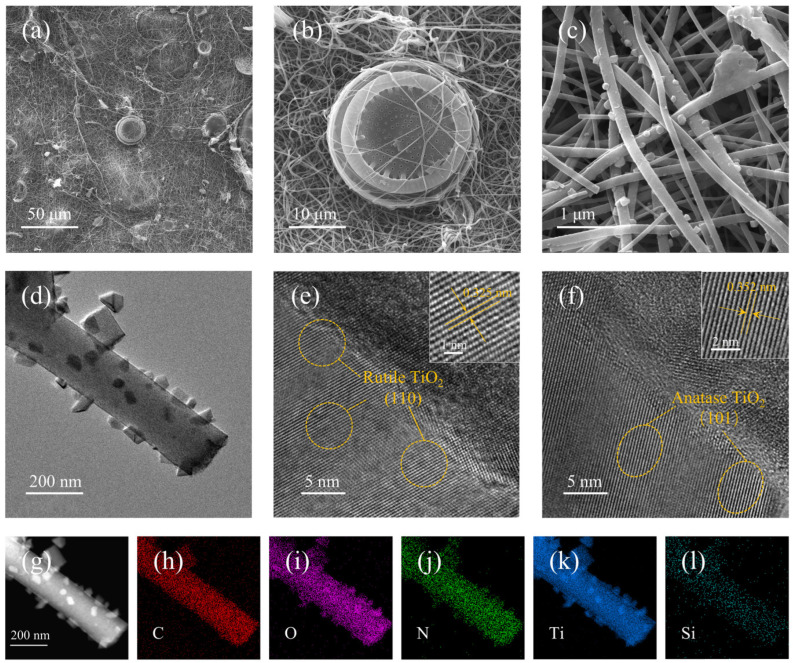
(**a**–**c**) SEM micrographs of NCNF/TiO_2_/DE at different magnifications, (**d**) TEM image, (**e**,**f**) high-resolution TEM images, (**g**–**l**) elemental mapping images of C, O, N, Ti, and Si.

After acid treatment, the impurity content on the surface of the diatomite was significantly reduced (Figure S1). As shown in Figure 2, the nanofiber in NCNF/TiO_2_/DE-800 interweaves to form a three-dimensional conductive network (Figure 2a–c), facilitating ion and electron transport and enhancing strain resistance.

After high-temperature carbonization, TiO_2_ nanoparticles were uniformly distributed within and around the nitrogen-doped CNF, and the surface of NCNF/TiO_2_/DE-800 was relatively rough. Numerous ultrafine metal nanoparticles were evenly distributed throughout the nanofiber, which provided more active sites for polysulfide adsorption. Diatomites were randomly dispersed, forming a disk-like porous structure with a diameter of about 25 μm, and partially wrapped by a few nanofibers, serving as a filler within the three-dimensional conductive network. This increased the loading surface area and contact area with the electrolyte, effectively absorbing polysulfides (Figure 2d). As shown in Figure 2e,f, high-resolution TEM (HRTEM) images clearly show regions corresponding to the rutile-phase TiO_2_ and anatase-phase TiO_2_. The lattice spacing of 0.325 nm corresponds to the (110) plane of the rutile-phase TiO_2_, and the lattice spacing of 0.352 nm corresponds to the (101) plane of the anatase-phase TiO_2_ [32,33].The EDS mapping indicates that the nanofiber primarily contains C, N, O, and Ti. C, Ti, and O elements are mainly concentrated in the nanofiber part, while N is uniformly distributed within the fibers, indicating good nitrogen doping. Si elements are primarily concentrated in the diatomite part and hence not prominently visible in the fiber part (Figure 2g–l).

**Figure 3:**
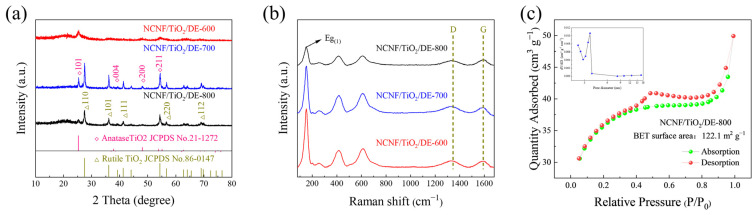
(**a**) XRD patterns of NCNF/TiO_2_/DE at 600 °C, 700 °C, and 800 °C. (**b**) Raman spectra of NCNF/TiO_2_/DE at 600 °C, 700 °C, and 800 °C. (**c**) Nitrogen adsorption/desorption isotherms and pore size distribution (PSD) curves of NCNF/TiO_2_/DE-800.

The thickness of the NCNF/TiO_2_/DE interlayer material is approximately 35 μm (Figure S2). An X-ray diffraction (XRD) analysis was performed to determine the phase composition of the NCNF/TiO_2_/DE composites (Figure 3a). When the carbonization temperature reached 600 °C, peaks at 25.3°, 37.8°, 48.0°, and 55.1° were observed in the XRD patterns of all three samples (NCNF/TiO_2_/DE-600, NCNF/TiO_2_/DE-700, NCNF/TiO_2_/DE-800), corresponding to the (101), (004), (200), and (211) planes of the anatase-phase TiO_2_ (JCPDS file number 21-1272). At 700 °C, additional peaks at 27.4°, 36.1°, 41.2°, 56.7°, and 69.8° were identified as the (110), (101), (111), (220), and (112) planes of rutile-phase TiO_2_ (JCPDS file number 86-0147), consistent with the HRTEM results [34]. Raman spectroscopy was used to analyze the composition of the material. Peaks at 144 cm^−1^, 399 cm^−1^, and 638 cm^−1^ match well with TiO_2_ [35,36,37]. Peaks at 1340 cm^−1^ and 1580 cm^−1^ correspond to the vibration modes of disordered carbon (D peak) and sp^2^-hybridized carbon atoms (G peak), indicating a typical graphite structure [26]. Since the intensity ratios of the D band to the G band (*I*_D_/*I*_G_) negatively correlate with graphitization, the *I*_D_/*I*_G_ ratios of the three materials are 1.17, 0.98, and 0.94 (Figure 3b), respectively, indicating an increase in disorder with an increasing carbonization temperature [38,39,40]. Additionally, the nitrogen absorption and desorption test curves of NCNF/TiO_2_/DE composites were investigated (Figure 3c). The N_2_ adsorption/desorption isotherm exhibits a non-overlapping hysteresis loop, indicating a mesoporous structure [41].

As shown in Table 1, the specific surface areas of NCNF/TiO_2_/DE-800, NCNF/TiO_2_/DE-700, and NCNF/TiO_2_/DE-600, which were determined via the BET method, were 122.1 m^2^ g^−1^, 80.3 m^2^ g^−1^, and 3.7 m^2^ g^−1^, respectively, with NCNF/TiO_2_/DE-800 having the largest specific surface area (Figure S3). The isotherm in Figure 3c belongs to class I/IV and presents H4 hysteretic curves when the relative pressure is *P*/*P*_0_ = 0.4~0.9. At very low pressures (*P*/*P*_0_ ≤ 0.084), NCNF/TiO_2_/DE-800 still exhibits a microporous structure that is beneficial for polysulfide adsorption. The inset of Figure 3c shows the pore size distribution (PSD), indicating mesopores ranging from 2 to 18 nm in NCNF/TiO_2_/DE-800, providing ample space for volume expansion. Therefore, NCNF/TiO_2_/DE-800 has a higher specific surface area and pore structure, providing more reaction sites and promoting the capture of polysulfides during electrochemical oxidation-reduction processes, thereby enhancing charge storage capacity [42,43,44].

**Figure 4:**
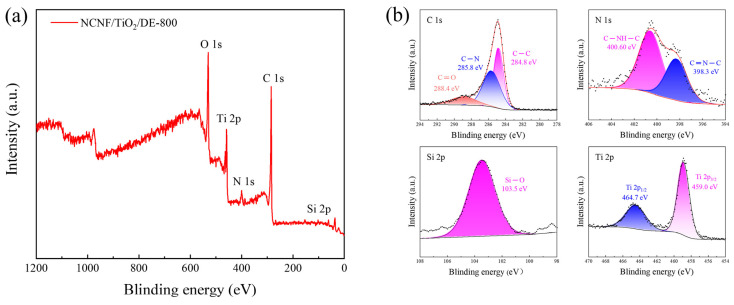
(**a**) XPS spectra of NCNF/TiO_2_/DE-800. (**b**) High-resolution XPS spectra of C 1s, N 1s, Ti 2p, and Si 2p.

X-ray photoelectron spectroscopy (XPS) was used to determine the elemental composition and the chemical states of each element. The chemical shifts refer to the binding energy changes in inner-shell electrons due to variations in the chemical environment around the atom, which are related to atomic valence, charge, and functional groups. Figure 4a shows the XPS survey spectrum of the NCNF/TiO_2_/DE-800 sample, containing C, Ti, O, N, and Si elements. Figure 4b presents the high-resolution XPS spectra for C 1s, N 1s, Si 2p, and Ti 2p. In the C 1s spectrum, peaks at 284.5, 285.8, and 289.1 eV correspond to C–C, C–N, and C=O [45,46,47]. The high-resolution N 1s XPS spectrum shows peaks at 398.3 eV and 400.6 eV, corresponding to pyridinic-N and graphitic-N, confirming successful nitrogen doping due to cyclization and dehydrogenation [48]. The high-resolution Ti 2p XPS spectrum shows peaks at 465.1 eV for Ti 2p_1/2_ and 459.3 eV for Ti 2p_3/2_ [49,50]. The high-resolution Si 2p XPS spectrum shows a peak at 103.5 eV, corresponding to the Si–O bond in diatomite [51].

From Figure S4, after the Li_2_S_6_ adsorption capacity test, the color of the solution in the vial containing NCNF/TiO_2_/DE-800 became clear and transparent after 12 h, while the solution in the vial containing NCNF/TiO_2_/DE-700 turned slightly yellow, and the solution in the vial containing NCNF/TiO_2_/DE-600 remained similar to the original Li_2_S_6_ solution. UV-Vis absorption tests of the supernatants showed that NCNF/TiO_2_/DE-600 exhibited the lowest absorbance, likely due to differences in porosity and changes in TiO_2_ crystal phases [52]. These results indicate that the NCNF/TiO_2_/DE-800 interlayer helps mitigate the shuttle effect of polysulfides during battery cycling.

**Figure 5:**
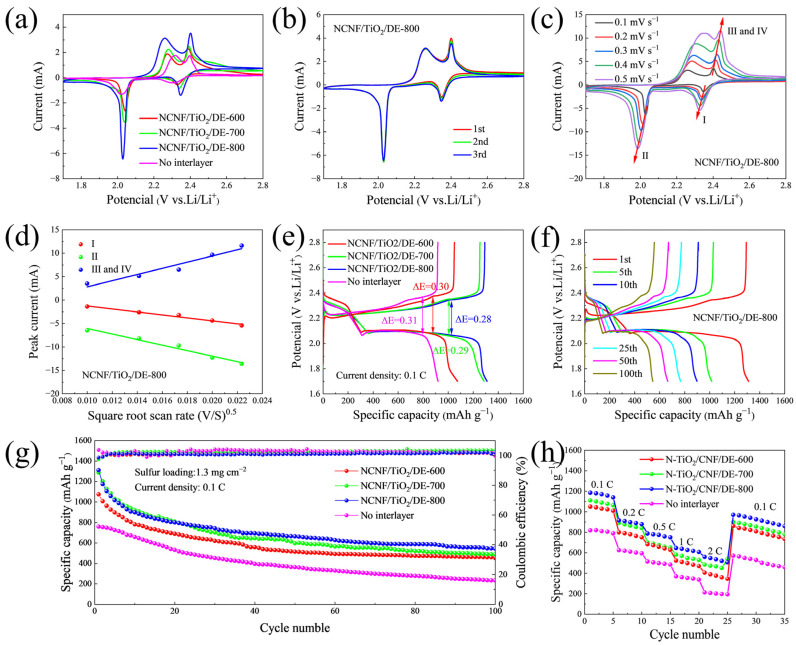
(**a**) CV curves of batteries with different intermediate layers at a scan rate of 0.1 mV s^−1^. (**b**) CV curve of the battery with NCNF/TiO_2_/DE-800 intermediate layer. (**c**) CV curves of the battery with NCNF/TiO_2_/DE-800 intermediate layer at scan rates ranging from 0.1 to 0.5 mV s^−1^. (**d**) Relationship between peak current values for anodic and cathodic processes and the square root of the scan rate obtained from CV measurements. (**e**) Initial charge–discharge curves of batteries with different intermediate layers at 0.5 C. (**f**) Constant current charge–discharge curves of the battery with NCNF/TiO_2_/DE-800 intermediate layer at 0.1 C over 1, 5, 10, 25, 50, and 100 cycles. (**g**) Cycling performance of batteries with different intermediate layers at 0.1 C over 100 cycles. (**h**) Charge–discharge curves of the battery with NCNF/TiO_2_/DE-800 intermediate layer at different current densities.

The improvement of the electrochemical performance of the coin battery by the functional intermediate layer is shown in Figure 5. Figure 5a shows the cyclic voltammograms (CV) of batteries with different interlayers in the potential range of 1.7–2.8 V at a scan rate of 0.1 mV s^−1^. Compared to other batteries, the battery with the NCNF/TiO_2_/DE-800 interlayer exhibited a significant positive shift in reduction peaks and a negative shift in oxidation peaks, with increased peak currents. This indicates that NCNF/TiO_2_/DE-800 can effectively adsorb polysulfides and accelerate their conversion reactions, thereby significantly enhancing the electrochemical performance. Notably, the NCNF/TiO_2_/DE-800 interlayer showed the smallest potential difference between the reduction and the oxidation peaks, indicating that the composite materials facilitate the nucleation and deposition of Li_2_S. Figure 5b shows two reduction peaks and one oxidation peak in the CV curve, attributed to the typical redox reactions of LiPSs in Li–S batteries, where the two reduction peaks represent S_8_^2−^→S_6_^2−^→S_4_^2−^ and S_4_^2−^→S_2_^2−^→Li_2_S, and the oxidation peak represents Li_2_S_2_/Li_2_S →S [53]. The redox potentials remained almost unchanged over three cycles, with overlapping CV curves, indicating excellent electrochemical reversibility and stability (Figure 5b and Figure S5) [54]. The lithium-ion diffusion coefficient, an important factor affecting reaction kinetics, was determined from CV curves at different scan rates (0.1~0.5 mV s^−1^) (Figure 5c and Figure S6). By studying the lithium-ion diffusion coefficient, we can understand the reaction kinetics inside the battery. Combining the peak current and the Randles–Sevcik equation [55,56]:

$$I_{p}=2.69\times 10^{5}n^{1.5}AD_{{Li}^{+}}^{0.5}v^{0.5}C_{{Li}^{+}}$$

where $I_{p}$ is the peak current, *n* is the number of electrons involved in the redox reaction, *A* is the cathode area (1.13 cm^2^ in this case), $D_{{Li}^{+}}$ is the lithium-ion diffusion coefficient, $C_{{Li}^{+}}$ is the lithium-ion concentration, and *v* is the scan rate. The half power of the scan rate *v* is linearly related to the peak current $I_{p}$, as shown in Figure 5d and Figure S6d–f. Table 2 compares the calculated diffusion coefficients of different batteries, indicating that the battery with the NCNF/TiO_2_/DE-800 interlayer has the highest $D_{{Li}^{+}}$. These data suggest that the three-dimensional (3D) conductive NCNF/TiO_2_/DE-800 material exhibits superior electrochemical performance, facilitates the accommodation of active materials, inhibits the formation of insulating layers, and accelerates lithium-ion diffusion [36,57].

Figure 5e shows the first constant-current charge–discharge curves of batteries with different interlayers at 1.7–2.8 V vs. Li/Li^+^ at 0.5 C current density. Each discharge curve exhibits two typical discharge platforms, with the high-potential platform indicating the reduction in S to long-chain LiPSs, and the low-potential platform indicating the further reduction in LiPSs to short-chain Li_2_S_2_/Li_2_S. These results align well with the previously described CV curves. To assess polarization, the potential difference (ΔE) between charge and discharge curves for batteries with blank PP separators, NCNF/TiO_2_/DE-600, NCNF/TiO_2_/DE-700, and NCNF/TiO_2_/DE-800 interlayers were calculated as 0.28 V, 0.29 V, 0.30 V, and 0.31 V, respectively (Figure 5e). Thus, the battery with the NCNF/TiO_2_/DE-800 interlayer is expected to have lower polarization and better reversibility. As shown in Figure 5f, the charge–discharge curves of the Li–S battery with the NCNF/TiO_2_/DE-800 interlayer at 0.1 C for the 1st, 5th, 10th, 25th, 50th, and 100th cycles. The charge–discharge curves remain nearly unchanged with increasing cycles, attributed to minimal electrode polarization during cycling. Meanwhile, Figure S7 shows constant current charge/discharge curves of the Li–S battery with other kinds of interlayer at 0.1 C for the 1st, 5th, 10th, 25th, and 100th cycles. This indicates that the battery with the NCNF/TiO_2_/DE-800 interlayer has a higher initial capacity, better reversibility, and smaller potential differences, confirming that NCNF/TiO_2_/DE-800 accelerates the reaction kinetics of LiPSs during charge–discharge cycles.

Figure 5g shows the cycling performance of coated separator batteries at 0.1 C current density. The first cycle discharge specific capacities of batteries with blank PP separators, NCNF/TiO_2_/DE-600, NCNF/TiO_2_/DE-700, and NCNF/TiO_2_/DE-800 coated separators are 919.6 mAh g^−1^, 1187.9 mAh g^−1^, 1287.4 mAh g^−1^, and 1311.1 mAh g^−1^, respectively. After 100 cycles, the battery with the NCNF/TiO_2_/DE-800 coated separator retains a discharge capacity of 547.2 mAh g^−1^, representing 41.7% of its initial capacity, higher than the blank PP (234.3 mAh g^−1^, 25.5%), NCNF/TiO_2_/DE-600 (460.6 mAh g^−1^, 38.8%), and NCNF/TiO_2_/DE-700 (488.6 mAh g^−1^, 37.9%). These results indicate that the NCNF/TiO_2_/DE-800 coated separator improves the cycling stability and performance of Li–S batteries due to the synergistic effect of porous materials and excellent adsorption and catalytic abilities towards LiPSs [58]. Additionally, Table S1 compares the battery performance with other studies, demonstrating that the NCNF/TiO_2_/DE-800 interlayer battery shows superior electrochemical performance.

At different current densities, the first cycle discharge specific capacities of NCNF/TiO_2_/DE-800 are 1186.1 mAh g^−1^, 916 mAh g^−1^, 788.2 mAh g^−1^, 646.5 mAh g^−1^, and 558.6 mAh g^−1^, respectively, higher than those of batteries with blank PP, NCNF/TiO_2_/DE-600, and NCNF/TiO_2_/DE-700 coated separators (Figure 5h). Additionally, when the current density is restored to 0.1 C, the capacity of the Li–S battery with the NCNF/TiO_2_/DE-800 coated separator recovers to 970.0 mAh g^−1^, with a rate recovery rate of 81.78%. Therefore, the battery with the NCNF/TiO_2_/DE-800 interlayer material exhibits superior cycling performance compared to batteries without an interlayer.

**Figure 6:**
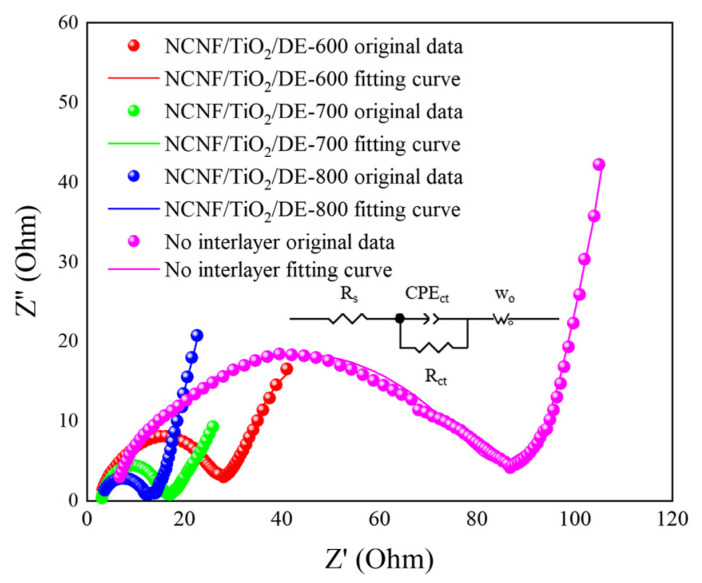
Nyquist plots of batteries with different intermediate layers.

Electrochemical impedance spectroscopy (EIS) tests provided insight into the charge-transfer kinetics within the battery [59]. EIS measurements were conducted over a frequency range of 10 mHz to 100 kHz. The intercept of the semicircle with the real axis at high frequencies represents the electrode and electrolyte resistance (R_s_), while the semicircle at high frequencies corresponds to the charge-transfer resistance (R_ct_) associated with the conversion of long-chain LiPSs to short-chain Li_2_S_2_/Li_2_S on the cathode. The Warburg (W_o_) impedance corresponds to the diffusion impedance of the lithium ions in the electrolyte. The equivalent circuit fitting yielded the resistance values listed in Table S2. The batteries with interlayers exhibited lower R_ct_ values compared to those without interlayers, indicating increased reduction reaction kinetics [60]. As shown in Figure 6, the NCNF/TiO_2_/DE-800 interlayer had the smallest charge-transfer resistance, suggesting that there were fewer Li_2_S_2_/Li_2_S deposits on the electrode surface. This indicates that NCNF/TiO_2_/DE-800 provides sufficient active sites for polysulfides, effectively accelerating the reaction kinetics and enhancing electrolyte penetration and lithium-ion diffusion.

## 4. Conclusions

In summary, this study designed an intermediate layer material for lithium–sulfur batteries by dispersing diatomite within the nitrogen-doped carbon nanofiber/titanium dioxide (NCNF/TiO_2_) three-dimensional network structure to accelerate the catalytic conversion of lithium polysulfides (LiPSs) in lithium–sulfur (Li–S) batteries. The incorporation of numerous TiO_2_ nanoparticles and nitrogen atoms, which were dispersed within the interwoven carbon nanofiber (CNF), facilitated the formation of a three-dimensional conductive network, characterized by abundant active adsorption sites, while diatomite occupied the interstices of the carbon nanofiber matrix. As an intermediate layer in lithium–sulfur batteries, this material exhibits excellent polysulfide capture capabilities, accelerates lithium-ion diffusion, and significantly reduces the “shuttle effect”. At 0.1 C, the NCNF/TiO_2_/DE-800-coated separator achieved a first-cycle discharge specific capacity of 1311.1 mAh g^−1^, significantly higher than the unmodified lithium–sulfur battery (919.6 mAh g^−1^). Under varying current densities, the NCNF/TiO_2_/DE-800 material demonstrated good electrochemical reversibility. Additionally, the presence of the NCNF/TiO_2_/DE-800 intermediate layer exhibited an excellent rate performance and a low charge-transfer resistance. This study validates the feasibility of NCNF/TiO_2_/DE-800 as a promising intermediate layer material for lithium–sulfur batteries, demonstrating the potential for advanced energy storage device design and fabrication.

## Figures and Tables

**Table 1:** Specific surface area and pore diameter of NCNF/TiO_2_/DE at 600 °C, 700 °C, and 800 °C.

Thermophysical Properties	NCNF/TiO_2_/DE-600	NCNF/TiO_2_/DE-700	NCNF/TiO_2_/DE-800
BET surface area(m^2^ g^−1^)	3.7	80.3	122.1
Pore volume(cm^3^ g^−1^)	0.0039	0.045	0.066
Average pore size(nm)	6.07	6.54	3.70
Median pore width (nm)	0.754	0.745	0.745

**Table 2:** Lithium-ion diffusion coefficients $D_{{Li}^{+}}$ for the batteries with different interlayers.

Cell	I (cm^2^ s^−1^)	II (cm^2^ s^−1^)	III and IV (cm^2^ s^−1^)
No interlayer	(1.24 ± 0.01) × 10^−10^	(4.71 ± 0.02) × 10^−10^	(5.69 ± 0.05) × 10^−10^
NCNF/TiO_2_/DE-600	(3.24 ± 0.11) × 10^−10^	(4.01 ± 0.43) × 10^−10^	(1.48 ± 0.04) × 10^−9^
NCNF/TiO_2_/DE-700	(5.31 ± 0.01) × 10^−10^	(5.38 ± 0.20) × 10^−10^	(1.71 ± 0.00) × 10^−9^
NCNF/TiO_2_/DE-800	(1.37 ± 0.01) × 10^−9^	(4.72 ± 0.04) × 10^−9^	(5.88 ± 0.12) × 10^−9^

## Data Availability

The original contributions presented in this study are included in the article/supplementary material. Further inquiries can be directed to the corresponding author.

## Supplementary Materials

The following supporting information can be downloaded at: https://www.mdpi.com/article/10.3390/ma17225615/s1. Figure S1: SEM images of the (a) primitive diatomaceous earth and (b) acidified diatomaceous earth; Figure S2: SEM image of a cross-section of NCNF/TiO_2_/DE-800 interlayer; Figure S3: Nitrogen adsorption–desorption isotherms and PSD curves of the (a) NCNF/TiO_2_/DE-600 and (b) NCNF/TiO_2_/DE-700; Figure S4: UV-vis spectra of different solutions; Figure S5: Cyclic voltammograms of the batteries with the different inter-layer materials; Figure S6: (a–c) Cyclic voltammograms of the batteries employing the different interlayer materials at various scan rates. (d–f) Peak current values for the anodic and cathodic processes versus the square root of the scan rates derived from CV; Figure S7: Constant current charge–discharge curves of the battery with (a) no inter-layer, (b) NCNF/TiO_2_/DE-600 interlayer, (c) NCNF/TiO_2_/DE-700 interlayer at 0.1C for the 1st, 5th, 10th, 25th, and 100th cycles; Table S1:Comparison of electrochemical performance of this work with previous work; Table S2: Resistance values of LSBs with different intermediate layers. References [61,62,63,64,65,66,67] are cited in the supplementary materials.
