# Supplementary material for: Design of Composite N-Doped Carbon Nanofiber/TiO2/Diatomite Separator for Lithium–Sulfur Batteries
Source: Materials (Basel). 2024 Nov 17;17(22):5615. doi: 10.3390/ma17225615 (PMC11595313; doi:10.3390/ma17225615)
Supplement: Supplementary file 1 [file materials-17-05615-s001.zip › materials-3299008-supplementary.pdf]

Supplementary Information

Design of Composite N-Doped Carbon Nanofiber/TiO2/Diatomite Separator for Lithium–Sulfur Batteries

Wenjie Xiao [1], Xiaoyu Wu [1], Yang Shu [1], Yitao Zha [2] and Sainan Liu [1,*]

[1] *School of Minerals Processing and Bioengineering, Central South University, Changsha 410083, China; xiaowenjie9574@163.com (W.X.)*

[2] *School of Materials Science and Engineering, Central South University, Changsha 410083, China*

[*] *Correspondence: lsn@csu.edu.cn*

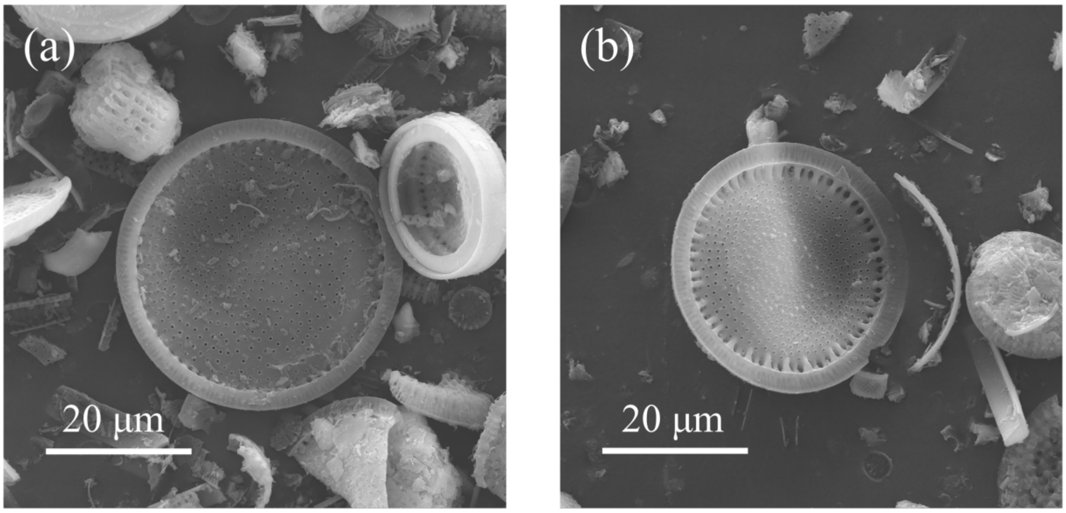

Figure S1. SEM images of the (a) Primitive diatomaceous earth and (b) Acidified diatomaceous earth.

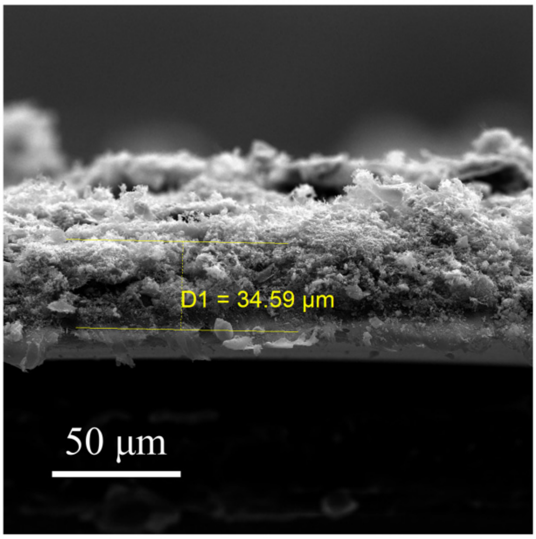

Figure S2. SEM image of a cross-section of NCNF/TiO_2/DE-800 interlayer.

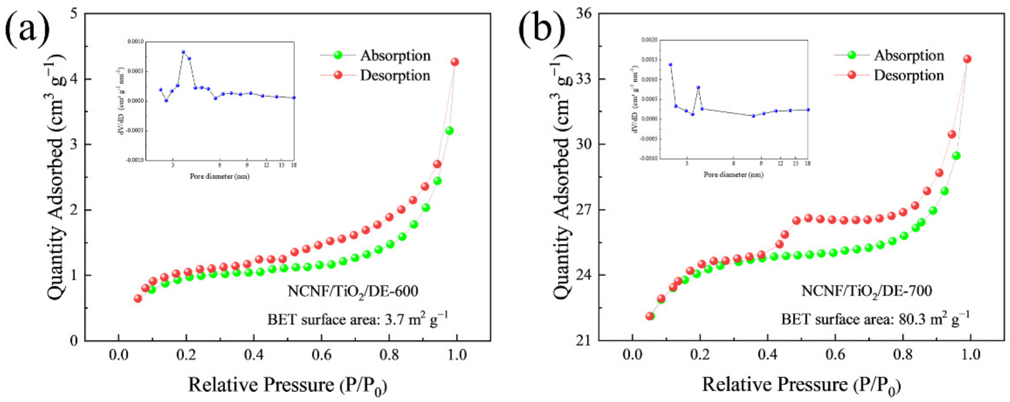

Figure S3. Nitrogen adsorption–desorption isotherms and PSD curves of the (a) NCNF/TiO_2/DE-600 and (b) NCNF/TiO_2/DE-700.

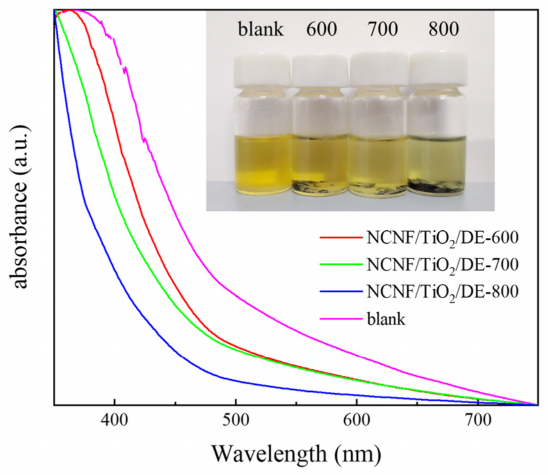

Figure S4. UV-vis spectra of different solutions.

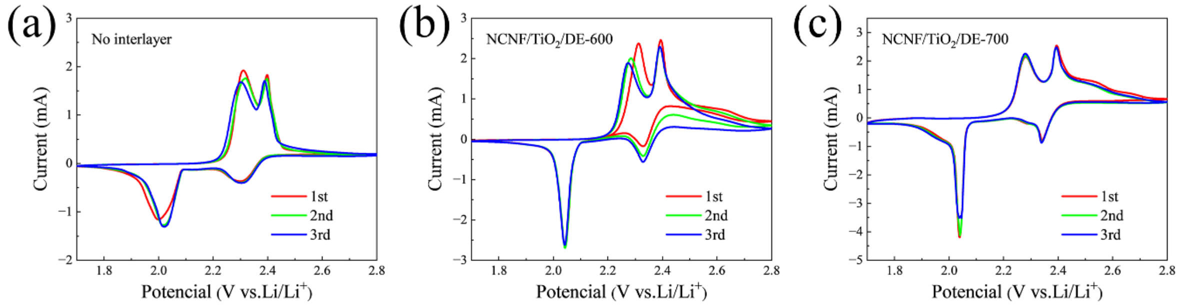

Figure S5. Cyclic voltammograms of the batteries with the different interlayer materials.

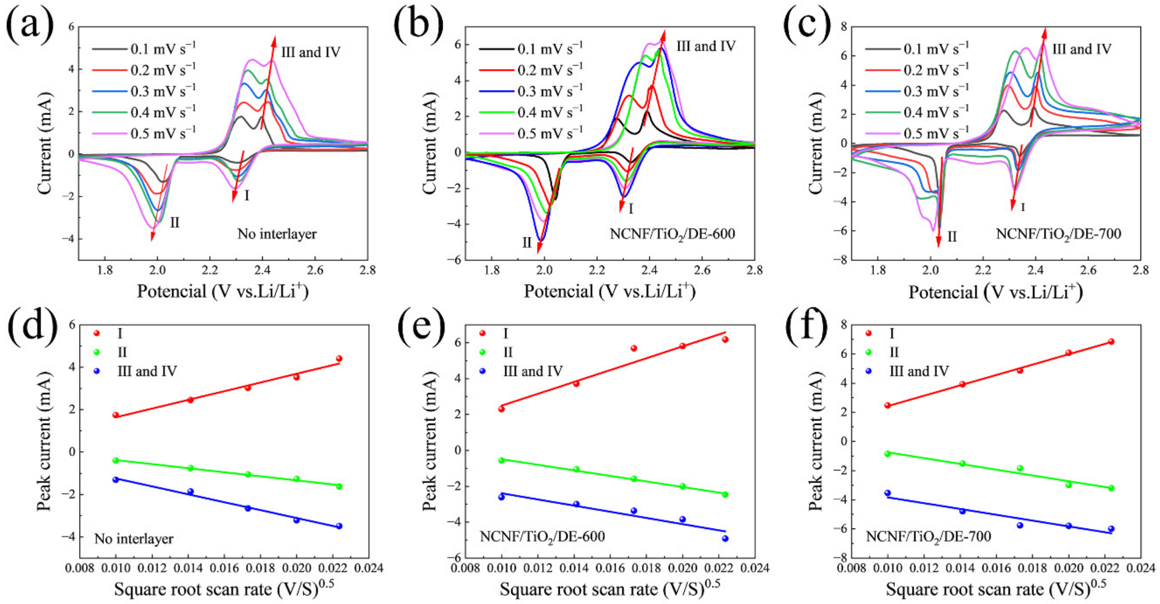

Figure S6. (a-c) Cyclic voltammograms of the batteries employing the different interlayer materials at various scan rates. (d-f) Peak current values for the anodic and cathodic processes versus the square root of the scan rates derived from CV.

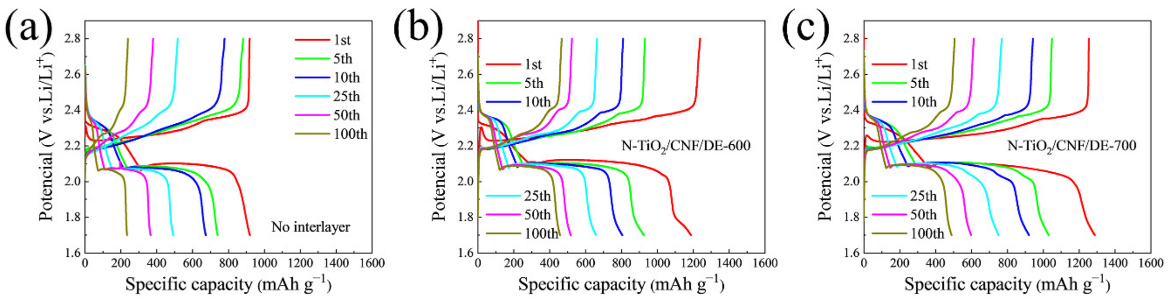

Figure S7. Constant current charge-discharge curves of the battery with (a) no interlayer、(b) NCNF/TiO_2/DE-600 interlayer、(c) NCNF/TiO_2/DE-700 interlayer at 0.1C for the 1st, 5th, 10th, 25th and 100th cycles.

Table S1. Comparison of electrochemical performance of this work with previous work.

**Cell**	**Current density (1 C=1675 mA g^{-1})**	**Cycle number**	**Initial capacity (mAh g^{-1})**	**Capacity decay ratio(%)**	***Ref.***
CeO_2/RGO	0.1C	100	1136	0.22	[1]
Ni-WS_2@rGO	0.2C	100	1160	0.45	[2]
RGO/TiO_2(B)	0.2C	100	1097	0.59	[3]
PPY/ZnO	0.2C	100	1194	0.52	[4]
TiO_2-NS-rGO/S	0.2C	100	1099	0.37	[5]
CAF	0.2C	100	1096	0.31	[6]
Nylon scale graphene	0.1C	20	1128	1.46	[7]
**NCNF/TiO_2/DE-800**	**0.1 C**	**100**	**1311**	**0.58**	**This work**

Table S2. Resistance values of LSBs with different intermediate layers.

Cell / Element	No interlayer		NCNF/TiO_2/DE-600		NCNF/TiO_2/DE-700		NCNF/TiO_2/DE-800	
	Value/Ω	Error/%	Value/Ω	Error/%	Value/Ω	Error/%	Value/Ω	Error/%
R_s	3.16	3.04	2.54	2.85	2.86	2.15	2.63	3.59
R_{ct}	79.7	0.51	24.23	1.25	11.99	1.32	9.43	1.42
W_o	32.23	9.97	38.27	8.44	9.19	9.83	7.01	7.04

References

[1] S. Wang, F. Gao, Y. Zhao, N. Liu, T. Tan, X. Wang, Two-Dimensional CeO_2/RGO Composite-Modified Separator for Lithium/Sulfur Batteries, Nanoscale Research Letters, 13(1) (2018) 377, https://doi.org/10.1186/s11671-018-2798-5.

[2] M.A. Al-Tahan, Y. Dong, A.E. Shrshr, X. Liu, R. Zhang, H. Guan, X. Kang, R. Wei, J. Zhang, Enormous-sulfur-content cathode and excellent electrochemical performance of Li-S battery accouched by surface engineering of Ni-doped WS_2@rGO nanohybrid as a modified separator, Journal of Colloid and Interface Science, 609 (2022) 235-248, https://doi.org/https://doi.org/10.1016/j.jcis.2021.12.035.

[3] P. Chen, Z. Wang, B. Zhang, H. Liu, W. Liu, J. Zhao, Z. Ma, W. Dong, Z. Su, Reduced graphene oxide/TiO_2(B) nanocomposite-modified separator as an efficient inhibitor of polysulfide shuttling in Li-S batteries, RSC ADVANCES, 10(8) (2020) 4538-4544, https://doi.org/10.1039/c9ra10185c.

[4] F. Yin, J. Ren, Y. Zhang, T. Tan, Z. Chen, A PPy/ZnO functional interlayer to enhance electrochemical performance of lithium/sulfur batteries, Nanoscale Research Letters, 13(1) (2018) 307, https://doi.org/10.1186/s11671-018-2724-x.

[5] S. Hong, Y. Han, K. Zhang, M. Wang, N. Cui, X. Du, Q. Li, Y. Huang, F. Jiang, K. Xie, TiO_2 Nanosheet-Redox Graphene Oxide/Sulphur Cathode for High-Performance Lithium-Sulphur Batteries, JOURNAL OF NANOSCIENCE AND NANOTECHNOLOGY, 20(3) (2020) 1715-1722, https://doi.org/10.1166/jnn.2020.16957.

[6] X. Chen, Y. Huang, J. Li, X. Wang, Y. Zhang, Y. Guo, J. Ding, L. Wang, Bifunctional separator with sandwich structure for high-performance lithium-sulfur batteries, Journal of Colloid and Interface Science, 559 (2020) 13-20, https://doi.org/https://doi.org/10.1016/j.jcis.2019.10.001.

[7] X. Ou, Y. Yu, R. Wu, A. Tyagi, M. Zhuang, Y. Ding, I.H. Abidi, H. Wu, F. Wang, Z. Luo, Shuttle Suppression by Polymer-Sealed Graphene-Coated Polypropylene Separator, ACS Applied Materials & Interfaces, 10(6) (2018) 5534-5542, https://doi.org/10.1021/acsami.7b17251.
